# The impact of suicide prevention training for nursing assistant students: Knowledge and willingness to intervene

**DOI:** 10.1371/journal.pone.0323169

**Published:** 2025-05-07

**Authors:** Mette Valdersdorf Jensen, Stine Bojen Aagaard Frandsen, Katrine Ulrikke Madsen, Lena Rossen Østergaard, Christina Petrea Larsen, Erik Christiansen

**Affiliations:** 1 Centre for Suicide Research, Odense, Denmark; 2 Department of Regional Health Research, Faculty of Health Sciences, University of Southern Denmark, Odense, Denmark; 3 Centre for Suicide Prevention, Odense, Denmark; Caleb University, NIGERIA

## Abstract

Nursing assistants frequently encounter individuals at risk of suicidal behavior in various healthcare settings. This study aims to assess the impact of suicide prevention training materials on nursing assistant students’ knowledge of, and willingness to intervene in suicidal behavior. Pre- and post-training assessments were conducted using the Revised Facts on Suicide Quiz and the Willingness to Intervene against Suicide Questionnaire measuring students’ knowledge of and willingness to intervene in suicidal behavior. Repeated measures analyses were used to assess changes before and after the use of teaching materials. Students significantly improved overall scores on the Revised Facts on Suicide Quiz (from a pretest mean of 9.55 ± 2.97 to a posttest mean of 13.28 ± 2.51, (p < .001) and the Willingness to Intervene against Suicide Questionnaire (from a pretest mean of 85.63 ± 8.22 to a posttest mean of 92.28 ± 8.93, (p < .001.). Attitudes were not a significant predictor of intention to intervene. These findings underscore the importance of training nursing assistants in suicide prevention as a means of early intervention of suicide behavior. Fostering a proactive attitude and willingness to intervene is crucial for nursing assistants in early identification and intervention in cases of suicidal behavior within healthcare settings. Finally, the results underscore the importance of targeted suicide prevention curricula in social and health-related vocational programs.

## Introduction

Suicide and its associated behaviors remain a significant public health problem, with over 700,000 suicide-related deaths every year [1]. Suicidal behavior encompasses a wide spectrum of behaviors, including suicidal thoughts, suicide attempts, and suicide [2]. The risk factors have been found to be of social, demographic, and economic character [3], with psychiatric diagnoses like borderline personality disorder, addiction, depression, bipolar disorder and schizophrenia posing the highest risk [4,5].

Various strategies and initiatives to reduce and prevent suicide and suicidal behavior have been identified and are continually developed and evaluated (such as means restriction, public awareness and screening programmes) [6]. Among these strategies is an increasing focus on education and training of social and healthcare professionals. Targeted training and education for healthcare professionals have been demonstrated to be effective in increasing knowledge and improving attitude towards suicide prevention [7], which is likely to have a positive impact on suicide prevention efforts [8]. The importance of training and education of health care professionals is underscored by the fact that persons who die from suicide often had contact with a health care professional prior to their death [9], highlighting the pivotal role that social - and healthcare professionals can play in early suicide prevention.

Prior training efforts developing and evaluating suicide prevention training, have primarily targeted nurses [7], medical students [10] and other health care professionals at undergraduate [11] and postgraduate level [12]. Yet, nursing assistants are a group of health care professionals who frequently interact with persons at risk of suicidal behavior, e.g., in primary care or emergency settings [13,14]. Despite this, the majority of nursing assistants employed in psychiatry or primary care have never received systematic training in suicide prevention [14], and consequently lack the knowledge, skills, and confidence necessary to assess and prevent situations with where individuals exhibit suicidal behavior [13]. To our knowledge, there is currently little to no research specifically focusing on suicide prevention training for health-related vocational students, such as nursing assistants. This is despite the likelihood that these groups will come into contact with people with suicidal behavior and play crucial roles within the healthcare workforce. Nursing assistants can potentially play a crucial role in early suicide prevention, especially in home care settings, where they have frequent, direct contact with patients. Although specific research on nursing assistants and suicide prevention is limited, training nursing assistants to recognize signs of distress can potentially enhance early detection and intervention, positioning them as key players in suicide prevention.

Evaluation of suicide prevention training efforts often prioritizes the assessment of knowledge, attitudes and skills [15–17] Some studies have extended this evaluation to include participants’ intention to intervene against suicide, employing the Theory of Planned Behavior (TPB) as a framework [15]. TPB provides a structured understanding of how attitudes, subjective norms, and perceived behavioral control influence behavioral intentions [16]. In the context of suicide prevention, TPB can potentially help to highlight the factors that shape individuals’ willingness to intervene (such as other people’s expectations, and their confidence in acting in certain situations). Given the frequent interactions of nursing assistant students with individuals at risk of suicide, employing a survey grounded in TPB could help to gain valuable insights into their readiness and willingness to intervene in suicide-related situations. As such, a part of the evaluation in the present study is using “The Willingness to Intervene Against Suicide Questionnaire”, which is based on the TPB.

This study aims to help bridge the research gap in suicide prevention resources specifically designed for nursing assistants. By providing nursing assistants students with targeted teaching materials about suicide and suicide prevention there is potential to increase their knowledge and improve their willingness to intervene in the care for patients with suicidal behavior. Given nursing assistants frequent interaction with individuals at risk of suicidal behavior, this presents a valuable opportunity for early intervention, which could contribute to suicide prevention efforts.

This study evaluates nursing assistants’ knowledge of and intention to intervene against suicide, pre- and post the use of targeted teaching materials.

## Materials and methods

This study involved an online pre – and post survey assessment of nursing assistant students’ knowledge of and willingness to intervene in suicide and suicide prevention following the use of teaching materials.

### Participants

The participants were first year nursing assistant students from two Danish social – and healthcare schools in the Region of Southern Denmark.

### Materials

The content of the teaching materials was developed based on a workshop with a group of nursing assistant students, who had been in their first clinical practice in primary care. The students were asked 1) *What they wish they had known about suicide and suicide prevention prior to their first clinical internship*, and 2) *What teaching format they preferred*. Based on the students’ responses, the materials were divided into four different modules: introduction, knowledge and prevention, tools and techniques and follow-up and support options. Each module contained an overview intended for the teacher, providing them with recommended literature and ideas for planning a teaching lesson based on the content of the module. Among other things, the teaching materials consisted of literature, powerpoint shows, and skill acquisition in the form of cases and classroom activities. Additionally, five educational movies were produced, dealing with different themes in relation to suicide and suicide prevention. This included a movie about a previous psychiatric patient talking about suicidal thoughts, a movie with a person bereaved by suicide, and a movie about resources and referral points specifically focusing on the role of nursing assistants.

The format of the teaching materials was based on students’ responses from the workshop as well as recommendations from the literature on the benefits of blended learning [18] (i.e., the materials were sought to be a combination of self-study, classroom teaching, activities and movies). The materials were freely available at the school’s online platforms, where the responsible teachers accessed the teaching materials through their own platform. They could freely pick the materials they deemed relevant and useable for their specific group of students.

### Data collection

The social – and health schools were invited to participate in the study if they 1) were based in the region of southern Denmark and 2) the teachers agreed to apply the available teaching materials. The schools were invited to participate in the survey pre – and post the teaching and could freely use the materials even if they declined to complete the survey.

The survey was completed by nursing assistant students from two different social – and health schools in the region of southern Denmark before and after the use of the teaching materials. The pre-survey was completed by students on January 9^th^, 2023 (first school) and February 8th, 2023 (second school). The post-survey was completed by the students September 13^th^, 2023 (first school) and October 16^th^, 2023 (second school). The students were eligible to participate in the post-survey if: they had responded to the pre-survey, participated in classes where the teaching materials had been used, and had not been in their psychiatric clinical internship. The latter eligibility criteria was established to prevent the students from being enrolled in other suicide prevention training in their psychiatric internship. Although the pre-post design of the study cannot definitively determine the intervention’s impact, the inclusion of specific eligibility criteria was intended to minimize the potential influence of other teaching materials and sessions on the students’ survey outcome. If completing the survey students were offered to enter into a prize draw to win two cinema tickets.

The survey consisted of items from two validated questionnaires that were translated into Danish following best-practice translation guidelines, and with the acceptance from the original developers [19]. Twenty-one of the 36-item Revised Facts on Suicide Quiz (RFOS) [20] were included to assess the students’ level of knowledge about suicide, as well as their possible misconceptions about suicide. Fifteen items were omitted as they were country-specific to the United States (e.g., items related to firearms restriction in relation to suicide), and thus not relevant in a Danish context. The response formats for the items are in a *true, false, don’t know* format, or in multiple-choice format.

Table 1 below lists two examples of items from the RFOS along with their corresponding response categories.

**Table 1:** Example of items and response category from Revised Facts on Suicide Quiz.

Item	Response category
*People who talk about suicide rarely take their own lives*	True/ False/Do not know
*Men kill themselves in numbers X than those for women*	Similar to/Higher than/Lower than

The Willingness to Intervene Against Suicide Questionnaire (WIS) [21] was included to assess the students’ willingness to intervene against suicide. The questionnaire is based on the Theory of Planned Behavior (TPB) [22]. According to the TPB individuals’ intention to engage in a particular behavior is influenced by three main factors: their attitude toward the behavior, subjective norms related to the behavior, and their perceived behavioral control [23]. In simpler terms, TPB suggests that whether someone intends to do something or not depends on how they feel about the behavior, what they think others expect of them regarding that behavior, and how much control they perceive they have in performing the behavior.

The WIS consists of four different subscales with 42 items in total. The subscales are *attitude, subjective norms, perceived behavioral control* and *intention*. The items in the attitude scale relate to personal views on providing support. Subjective norms items explore the impact of societal approval, while perceived behavioral control items focus on the ease or difficulty of taking action. Items in the intention subscale address specific actions the respondent is willing to take to intervene.

Two items were omitted from the attitude subscale in the survey due to inequivalence between the original item and the translated item. Table 2 lists the subscales with an example of an item and their respective response category.

**Table 2:** Example of items and response categories from The Willingness to Intervene Against Suicide Questionnaire Subscales.

Subscale	Item	Response category
Attitude	*Intervening when someone is suicidal would be.*	5-point scale with adjective pairs, e.g., *Scary – Not scary*
Subjective norms	*Most people like me would talk to someone who may be suicidal.*	5-point Likert scale ranging from 1 (*strongly disagree*) to 5 (*strongly* *agree*)
Perceived behavioral control	*I am confident in my ability to discuss suicide with the suicidal person*	5-point Likert scale ranging from 1 (*strongly disagree*) to 5 (*strongly agree*)
Intention	*Express my concern for someone who is suicidal to a professional is.*	5-point Likert scale ranging from 1 (*not likely at all*) to 5 (*extremely likely*)

Demographic items were added to the survey assessing the participants’ gender and age, their previous experience with training in suicide prevention, as well as their experience with individuals who have attempted suicide or died by suicide.

The survey was hosted in SurveyXAct, where the students accessed the survey through an online link on their computer or tablet. The students were presented with a participant information page about the project and were asked to give consent prior to completing the survey. The consent was given by the students’ ticking a box. The students were required to complete the survey unassisted, however a researcher from the project group was present in the classroom when the students completed the survey to solve any queries relating to the survey questions.

### Ethics statement

No ethical approval was required for the study, as advised by the Research Ethics Committee of University of Southern Denmark. The survey adhered to ethical principles, ensuring voluntary participation, confidentiality, and anonymity. The data from the survey were safely stored in accordance with guidelines from University of Southern Denmark.

### Data analysis

The data were imported into Stata/BE 18 for analysis. For the RFOS scale, responses were dichotomized into correct and incorrect responses and “do not know” responses were coded as missing. For the WIS questionnaire, the items were coded from negative to positive side (i.e., higher number indicates a higher endorsement). Some items were reverse coded. Cronbach alpha values were calculated for both surveys pre and post to assess the internal reliability of the scales.

For demographic variables, means and standard deviations were calculated for continuous variables, and n (%) for categorical variables. To visually inspect the distribution of the data, histograms were created for the scores on RFOS and all subscales on the WIS questionnaire. Shapiro-Wilk tests were performed to test the normality of the data. Levene’s test was performed to assess the equality of variance. All assumptions were met.

Correlation analyses were conducted to assess the linear relationship between the variables in the WIS questionnaire on the pre – and post data individually.

T-tests were used to compare the means of the RFOS and the WIS scores between the pre and the post group of students. T-tests were also conducted for each individual item in the WIS questionnaire. Cohen’s d was calculated for effect size [24].

Regression analyses were used to examine the impact of attitude, subjective norms and perceived behavioral control on the student’s intention to intervene (the WIS questionnaire). Simple linear regression and residual analyses were performed for pre and post data individually. Following this, multiple linear regression analyses were performed for pre – and post data individually. Results were deemed statistically significant at *p* < 0.05.

Data is reported in accordance with the Defined Criteria To Report INnovations in Education (DoCTRINE) reporting checklist [25].

## Results

### Cronbach alpha values

As can be seen in Supporting information S1 Table, the Cronbach alpha values for the *Willingness to intervene against suicide questionnaire* subscales ranged from poor (*0.67* on the intention to intervene scale) to good (*0.85* on the perceived behavioral control scale) in the pre-test. In the post-test values ranged from moderate (*0.74* on the attitudes scale) to good (*0.85* on the subjective norms scale) in the post-test. For the two subscales in the *Revised Facts on Suicide Quiz questionnaire* both pre- and post-values were of poor reliability.

### Students’ characteristics

As can be seen in Table 3 below, 89 nursing assistant students participated in the pre-measurement. Of these, 68 participated in the post-measurement. The majority of participants were female (86%), with an average age of 33 ± 13 years, and the highest representation in the age group 37–60. The vast majority of the students had not received prior training in suicide prevention (85%). Slightly under one-third of the students had previously worked with individuals exhibiting suicidal behavior (32%), while the majority had not. More than half of the students reported personal encounters with individuals displaying suicidal behavior (56%). Two third of the students knew one or more individuals who had died from suicide (41%), and more than half knew one or more individuals who had attempted suicide (53%). In the group of 68 students participating both in the pre and the post measurements, the majority of participants were female (82%). The average age was 34 ± 13 years, with the highest representation of students in the age group 37–60 (41%). Students not completing both the pre – and post were all female, and the biggest drop-out was in the younger age groups.

**Table 3:** Demographic characteristics, training and experience with suicidal behaviour among nursing assistant students.

	*Pre only N* = 89	*Pre and post N = 68*
**Sex, n (%)**		
Female	77 (86.52)	56 (82.35)
Male	12 (13.48)	12 (17.65)
**Age, mean (SD** ^1^ **), range**	32.68 (12.65), 17-59	34.29 (12.98), 18-60.
**Age groups, n (%)**		
Age group 1 (17–23)	30 (33.71)	22 (32.35)
Age group 2 (24–36)	28 (31.46)	18 (26.47)
Age group 3 (37–60)	31 (34.83)	28 (41.18)
**Have you previously received training in suicide prevention, n (%)**		
Yes	9 (10.11)	62 (91.18)
No	76 (85.39)	3 (4.41)
Do not know	4 (4.49)	3 (4.41)
**Have you previously worked with persons with suicidal behavior, n (%)**		
Yes	29 (32.58)	25 (36.76)
No	58 (65.17)	38 (55.88)
Do not know	2 (2.25)	5 (7.35)
**Have you experienced persons you believed had suicidal behavior, n (%)**		
Yes	50 (56.18)	34 (50.00)
No	31(34.83)	29 (42.65)
Do not know	8 (8.99)	5 (7.35)
Do you know persons who have attempted suicide or who have died from suicide, n (%)*		
Yes, knows one or more who have died from suicide	37 (41.57)	35 (51.47)
Yes, knows one or more who have attempted suicide	48 (53.93)	29 (42.65)
No	19 (21.35)	16 (23.53)
Do not know	2 (2.25)	3 (4.41)

^1^Standard deviation, *Students could give more than one answer.

### Correlation analyses

The correlation matrices for variables showed shifts in associations pre – and post intervention. Pre-intervention, attitude towards suicide prevention (AT) correlated moderately (0.37) with intention to intervene (INT). Subjective norms (SN) exhibited a stronger correlation (0.51), and perceived behavioral control (PBC) showed a moderate correlation (0.48). Post-intervention, the correlation between attitude and intention weakened (0.23), while the association between subjective norms and intention strengthened (0.62). Perceived behavioral control showed a strong correlation (0.62) with intention. Results of the correlation analyses are shown in Table 4.

**Table 4:** Correlation of attitudes of suicide prevention and subjective norms, percieved behavioural control and intention to intervene: Pre- and posttest.

	AT	SN	PBC	INT
**Pretest**				
AT	1.00			
SN	0.42	1.00		
PBC	0.27	0.41	1.00	
INT	0.37	0.51	0.48	1.00
**Posttest**				
AT	1.00			
SN	0.26	1.00		
PBC	0.31	0.41	1.00	
INT	0.23	0.62	0.62	1.00

AT: Attitude, SN: Subjective norms, PBC: Perceived Behavioral Control; INT: Intention to intervene,

### T-tests

The t-tests of overall scores on the RFOS and WIS questionnaire revealed significant changes, pre – and post the intervention. The results of t-tests for the overall scores can be seen in Table 5. The t-tests of individual items of the WIS scale showed a majority of significant changes on the items relating to *Perceived behavioral control* (PBC) and *Intention to intervene* (INT). Results of the t-tests for individual items on the WIS scale can be seen in Supporting information S2 Table.

**Table 5:** T-Test on differences in the willingnes to intervene and revised facts of suicide quiz among nursing assistants students: Pre-and posttest.

Scale	Pretest (n* *= 89)		Posttest (n* *= 68)		df	95% Confidence Interval		t	*P*-value	Cohen’s d*
	Mean	SD	Mean	SD		Lower	Upper			
WIS^1^	85.63	8.22	92.28	8.93	6.65	3.93	9.37	4.84	<.001	1.11
AT^2^	41.55	8.02	43.07	8.04	1.52	-1.03	4.08	1.18	0.12	0.19
SN^3^	34.55	4.96	35.96	5.51	1.41	-0.25	3.06	1.68	0.05	0.27
PBC^4^	55.79	9.14	62.34	7.71	6.55	3.83	9.27	4.76	<.001	0.77
INT^5^	74.79	6.95	80.88	7.44	6.10	3.82	8.38	5.28	<.001	0.85
RFOS^6^**	9.55	2.97	13.28	2.51	3.73	2.84	4.61	8.31	<.001	1.34

df: Difference in means; *Effect size: small (d = 0.2), medium (d = 0.5), and large (d ≥ 0.8).

^1^WIS = Overall score on Willingness to Intervene Against Suicide Questionnaire,

^2^AT = Attitude,

^3^SN = Subjective Norms,

^4^PBC = Perceived Behavioral Control,

^5^INT = Intention,

^6^RFOS = Overall score on Revised Facts on Suicide Quiz.

**Score indicate number of correct answers. Score can range from 0–21.

#### Revised Facts on Suicide Quiz (RFOS).

The students’ overall score on the RFOS exhibited a significant change following the intervention, with students showing an increase from a pretest mean of 9.55 ± 2.97 to a posttest mean of 13.28 ± 2.51, (p < .001). The effect size (Cohen’s d) was 1.34, indicating a large effect size.

#### Willingness to Intervene against Suicide Questionnaire (WIS).

The students’ overall score on the WIS questionnaire had a significant increase from a pretest mean of 85.63 ± 8.22 to a posttest mean of 92.28 ± 8.93, (p < .001.). The effect size (Cohen’s d) was 1.11, indicating a large effect size.

#### Subscale: Attitude.

The attitude subscale did not show a statistically significant change post-intervention, with a p-value of 0.12. The mean increased slightly from 41.55 ± 8.02 to 43.07 ± 8.04. Of 12 items in the attitude subscale, 2 single items were statistically significant, with a small to medium effect size (0.19).

#### Subscale: Subjective norms.

Similarly, the subjective norms subscale did not exhibit a significant change, as p-value was 0.05. The mean increased from 34.55 ± 4.96 to 35.96 ± 5.51. Of 9 items in the subjective norms’ subscale, 2 single items were statistically significant, with a medium effect size (0.27).

#### Subscale: Perceived behavioral control.

The perceived behavioral control subscale demonstrated a significant improvement, showing an increase from a pretest mean of 55.79 (SD = 9.14) to a posttest mean of 62.34 ± 7.71, (p < .001.). The effect size (Cohen’s d) was 0.77, indicating a medium to large effect (0.77).

Of 17 items in the perceived behavioral control subscale, 9 single items were statistically significant, with a medium to large effect size. Particularly the item with the largest effect size was *“I have access to suicide intervention resources”* (d = 1.00).

#### Subscale: Intention to intervene.

The intention to intervene subscale also displayed a significant improvement, with participants showing an increase from a pretest mean of 74.79 ± 6.95 to a posttest mean of 80.88 ± 7.44, (p < .001). The effect size (Cohen’s d) was 0.85, indicating a large effect.

Of 20 items in the intention to intervene subscale, 12 single items were statistically significant, with a medium to large effect size. Particularly the item with the largest effect size was “*Ask the person if they are having suicidal thoughts or feelings”* (d = 1.02)*.*

#### Regression analyses.

As seen in Table 6 of simple linear regression analyses, attitudes (β = 0.32), subjective norms (β = 0.71), and perceived behavioral control (β = 0.36). were all significant predictors of intention to intervene in the pre-test analysis. Post-intervention, attitude (β = 0.21) became insignificant, where subjective norms (β = 0.84), and perceived behavioral control (β = 0.59) remained significant.

**Table 6:** Simple linear regression analyses for attitude, subjective norms and perceived behavioral control on intention to intervene.

	AT			SN			PCB		
	α	β	95% CI	α	β	95% CI	α	β	95% CI
INT pretest	61.42	0.32*	0.15; 0.49	50.19	0.71*	0.45; 0.97	54.58	0.36*	0.22; 0.50
INT posttest	71.88	0.21	-0.01; 0.43	50.80	0.84*	0.58; 1.10	43.82	0.59*	0.41; 0.78

*Statistically significant coefficients.

INT: Intention to intervene; AT: Attitude; SN: Subjective norms; PBC: Perceived Behavioral Control.

The multiple linear regression analysis was conducted to examine the associations between attitude (AT), subjective norms (SN), perceived behavioral control (PCB), and the intention to intervene against suicide behavior (INT) pre – and post the educational material. As seen in Table 7 of the pretest model, attitude (β = 0.14) was not a significant predictor of intention to intervene, while subjective norms (β = 0.45) and perceived behavioral control (β = 0.23) were positive significant predictors. Post-intervention, attitude (β = -0.02) remained insignificant, while subjective norms (β = 0.60) and perceived behavioral control (β = 0.43) remained significant predictors of the intention to intervene, with an increase in their positive association.

**Table 7:** Multiple linear regression for attitude, subjective norms and perceived behavioral control on intention to intervene.

		AT		SN		PCB	
	α	β	95% CI	β	95% CI	β	95% CI
INT Pretest	40.77	0.14	-0.03; 0.30	0.45*	0.16; 0.77	0.23*	0.09; 0.38
INT Posttest	33.81	-0.02	-0.19; 0.14	0.60*	0.35; 0.85	0.43*	0.24; 0.61

*Statistically significant coefficients; INT: Intention to intervene; AT: Attitude; SN: Subjective norms; PBC: Perceived Behavioral Control.

## Discussion

This study assessed nursing assistant students’ suicide knowledge and willingness to intervene against suicide before and after the use of flexible teaching materials on suicide prevention. The results showed a significant improvement in students’ overall scores on The Revised Facts on Suicide Quiz Questionnaire and the Willingness to Intervene against Suicide Questionnaire following the intervention. Furthermore, improvements were observed in the scores on the two Willingness to Intervene against Suicide Questionnaire subscales—perceived behavioral control and intention to intervene— after the intervention. Notably, the study found that attitude was not a significant predictor of students’ intention to intervene. Instead, the subscales of subjective norms and perceived behavioral control were identified as significant predictors of students’ intention to intervene both before and after the intervention.

Overall, these results highlight that nursing assistant students’ knowledge about suicide and suicide prevention as well as their willingness to intervene increases with a large effect, even when teaching materials are flexible and provided over a short period of time.

Previous studies with a similar design have also found increases in knowledge following short-duration teaching materials on suicide prevention for health care professionals [26,27]. However, to our knowledge this is the first pre-and post-analysis of teaching materials specifically targeted nursing assistant students.

Saini et al. (2020) used a pretest questionnaire with 20 questions to assess knowledge, attitudes, and confidence in suicide identification and referral. Chan et al. (2009) combined pre- and post-test measures with focus group interviews to evaluate attitudes, knowledge, competence, and stress levels. While both Saini et al. and Chan et al. focused on more general healthcare professionals, our study specifically targeted nursing assistants, using materials tailored to their role. The incorporation of blended learning in the present study, with content like educational films, literature, and case-based activities designed based on student feedback, may have provided a more practical and engaging learning experience compared to the more traditional methods used in the other studies.

The correlation analyses suggest that the intervention had an impact on the relationships between the variables within the Willingness to Intervene against Suicide Questionnaire. The reduced correlation between attitude and intention to intervene from pre to post-test suggests that changes in attitude does not necessarily align with changes in intention to intervene. Similarly, the strengthened correlation between subjective norms and intention to intervene, and perceived behavioral control and intention to intervene post-test, highlight the potential influence of these factors on nursing assistant students’ willingness to intervene. This suggests that prioritizing teaching materials to focus on enhancing nursing assistant students’ perceptions of social expectations and their confidence in their ability to intervene effectively, could be pivotal in increasing their willingness to engage in suicide prevention activities.

The shift in attitudes is further highlighted in the regression analyses. Attitude was a significant predictor in the pre-test single regression analysis, but its significance diminishes in both the post-test single regression and multiple regression models. This suggests that the influence of attitude on intention to intervene is overruled by the other factors; subjective norms and perceived behavioral control.

The multiple regression analysis provides a more comprehensive understanding of these variables’ relationship with intention to intervene, highlighting their nuanced associations. While the students did not improve their attitude score significantly, the multiple regression analyses revealed that this did not have an impact on the students’ intention to intervene. Students increased their intention to intervene, even though attitude remained an insignificant predictor of their willingness to intervene. This finding implies that the students, even if they perceive actions related to suicide prevention as challenging or scary, are still willing to intervene when necessary. In other words, their intention to take action in suicide prevention scenarios is not necessarily deterred by any negative attitude they may hold. This finding is consistent with a previous study by Aldrich et al. using the Willingness to Intervene against Suicide Questionnaire, where attitude alone was not a significant predictor of intention to intervene [22]. However, a recent study from the same author analysed the attitude variable in two dimensions by focusing on items relating to attitude about how intervening would impact the suicidal person (e.g., beneficial or not beneficial) and attitude about how intervening would impact the student themselves (e.g., scary or not scary), and found that the first dimension was a significant predictor of intention to intervene [28]. It suggests that individuals may be more inclined to intervene if they perceive it as beneficial, despite feeling fearful of the situation. While this distinction was not explicitly addressed in the present study, it underscores the importance of focusing education and training at promoting positive attitude regarding the value of intervening, even when feeling apprehensive

In a broader perspective these findings can be linked to other behavioral theories, such as th Social Cognitive Theory (SCT) and Self-Determination Theory (SDT). SCT emphasizes the role of self-efficacy, suggesting that individuals are more likely to engage in behavior if they believe they can perform it (Bandura 2004). Students may have increased their intention to intervene because they felt capable of doing so, even if their attitudes toward the task did not change. Similarly, SDT highlights that intrinsic motivation and a sense of autonomy can drive behavior, even with negative attitudes (Deci & Ryan, 2000). In this context, students’ increased intention to intervene may reflect their perceived competence, belief in their ability to act, and a sense of social responsibility, despite initial apprehension.

When looking at the specific items from the willingness to intervene against suicide questionnaire which showed significant improvement after the intervention (see S2 Table), it can be inferred where the teaching materials were primarily focused. In terms of perceived behavioral control and intention to intervene, the items that saw improvement related to students’ confidence, skills, and practical steps in intervention scenarios (e.g., respectively *I do not know where to seek help for someone who is suicidal* or *Ask the person if they are having suicidal thoughts or feelings)*. This improvement might have arisen from the blended educational approaches of, e.g., practicing skills in suicide risk assessment and watching educational videos about available resources and referral options. This assumption is supported by a recent overview on the strengths and limitations of suicide prevention training studies, where mixed training approaches with a focus on skills acquisition is highlighted as more effective compared to single teaching modalities (i.e., only didactic lessons) [29]. The limited improvement in single items on the attitude and the subjective norm scores after the intervention similarly highlights a gap in the effectiveness of the teaching materials in these areas.

### Strengths and limitations

A key strength in this study is the focus on an understudied health care profession, and nursing assistant students’ potential role in the suicide prevention education and training area. The impact of education and training on nursing assistants’ knowledge of suicide prevention and their intention to intervene has not previously been studied, yet the changes in the outcomes were found to be significant. Another strength of the study is the development of teaching materials based on user-involvement from both teachers and students, and further that the teaching materials were comprised of blended learning and had flexible access for teachers. Additionally, the study included the careful translation and use of validated measures. These factors are highlighted as strengths for educational approaches specifically in suicide prevention training [29].

However, there are methodological limitations. The population sample in this study was based on convenience sampling, comprising only of a small number of nursing assistant students from the region of southern Denmark, This approach inherently reduces the representativeness of the sample, as it may not capture the diversity of perspectives, experiences, or characteristics found in broader populations. Moreover, the study utilized a two-sample design, which prevented us from tracking individuals over time or assessing their individual progress from pre-test to post-test. Finally, the majority of the sample were female. Other health care related vocational directions (and male students), might have other needs for training materials in suicide prevention, and this should be explored in other studies.

While the teachers opted to use the teaching materials on behalf of the class, the students self-selected to complete the survey. The voluntary nature of participation might have introduced a self-selection bias, where students opting to engage in the survey might differ systematically from non-participants (e.g., students participating might be more invested in the specific subject). Furthermore, the pre- and post-design poses inherent limitations. External factors, such as alternative learning opportunities or personal experiences between the pre- and post-tests, may have influenced the students’ responses. While efforts were made to mitigate this (i.e., by having the students respond to the post survey before their psychiatric internship), individual experiences could have had an impact on the internal validity of the study.

The varying Cronbach’s alpha values highlight important considerations for interpreting the findings. Subscales of the Willingness to Intervene Against Suicide questionnaire with good reliability (e.g., perceived behavioral control) provide robust insights, while those with poor reliability (e.g., intention to intervene) should be interpreted cautiously. The consistently poor reliability of the Revised Facts on Suicide Quiz questionnaire could reflect the difficulty level of the items, which may not align well with the knowledge levels of nursing assistant students. This mismatch might have led to inconsistent responses, emphasizing the need to adapt the survey in future studies to better suit the educational background of this group. Additionally, the omission of certain items from the RFOS questionnaire may limit comparability with other studies.

Finally, the present study only assessed changes in knowledge and intention to intervene, and not actual behavior change from the nursing assistant students. While the employed outcome measures of knowledge and intention to intervene are relevant and were measured with validated tools, it is crucial to find ways to assess if and how the created intention to intervene is implemented into clinical practice. Delaney et al (2022) [29] discuss the strengths and limitations of suicide prevention training studies, suggesting the incorporation of intermediate measures such as the number of patients referred to relevant suicide prevention clinics, the development of safety plans, or the completion of risk assessments to address this gap.

### Implications in practice

The findings from this study could potentially influence the development of future teaching materials for nursing assistant students. Specifically, a greater emphasis could be put on subjective norms and perceived behavioral control, by ensuring that teaching materials aim to strengthen students’ understanding of social influences and increase their confidence in intervening effectively. Regarding attitude, which were found to be a non-significant predictor of intention to intervene in this study, and the more detailed finding from Aldrich et al. [28] that attitude about how intervening would impact the suicidal person was a significant predictor of intention to intervene, perhaps emphasis should not primarily be placed on improving positive attitude towards intervention in general. Instead, it is understandable that students may perceive engagement in suicide prevention as overwhelming or frightening. Therefore, shifting the focus of attitude improvement within teaching materials from reducing fear of intervention to instead emphasizing instilling confidence in students regarding their capability to intervene effectively, even in situations where they may feel hesitant, could be the path forward.

Research on and development of training and education in suicide prevention for nursing assistant students and vocational education in general have been overlooked. Previous studies have focused on developing materials for nurses, doctors, or other social – and health related university students at undergraduate and postgraduate level [7,10–12]. Studies on suicide and suicide prevention in a vocational educational context are few and have mainly been focused on mapping suicidal behavior and ideations among the students [30,31], rather than the training and implementation of suicide prevention programs aimed at the students. This might be due to the fact that students in vocational education can have a high risk of suicidal behavior [30]. However, vocational students encompass diverse backgrounds and fields of study, with those in social and health care likely to encounter individuals with suicidal behavior during clinical practice. Moreover, training and educating peers of at-risk groups in suicide prevention, might also have a preventative effect. By enhancing their understanding of suicide behaviors and empowering them with effective coping strategies, peers within these groups could potentially play important roles in preventing suicide (e.g., by reaching out to people who might not seek help for suicidal thoughts and acting as a bridge to support and resources [32]. Therefore, developing and implementing targeted suicide prevention curricula in social and health-related vocational programs is warranted.

Finally, future research should employ larger, more diverse samples to enhance the generalizability of findings and consider the diverse needs of other vocational healthcare groups. Ideally, future studies should employ randomized control trials or quasi-experimental designs with a control group to mitigate self-selection bias and control for external factors. Future research in education and training on suicide prevention should incorporate measures to evaluate how observed changes in knowledge and intention translate into practice.

## Conclusion

The findings demonstrate the effectiveness of a short-duration educational intervention in improving nursing assistant students’ suicide knowledge and willingness to intervene and offers direction for development of future teaching materials for this purpose. These materials should acknowledge students’ concerns about preventing suicide while offering practical strategies and evidence-based tools to increase confidence and competence. To ensure effective implementation, the teaching materials should be developed collaboratively with students, educators, and healthcare professionals. These materials can then be integrated into existing programs and modules, tailored to the specific vocational curricula and the competencies of the instructors. By providing nursing assistant students with the right skills and confidence, tailored teaching materials can enhance their intention to intervene in suicidal behavior. The study further highlights the need for further research on suicide prevention training for vocational healthcare students, particularly through longitudinal studies assessing knowledge retention and the impact on clinical behavior. To strengthen the evidence base, we recommend conducting longitudinal studies to assess whether nursing assistant students apply the suicide prevention knowledge gained during training in real-world settings. These studies could focus on intermediate measures, such as the frequency and quality of suicide risk assessments, or the initiation of preventive actions like referrals and follow-up care. By tracking these behaviors over time, researchers can evaluate whether the training leads to tangible outcomes, such as improved intervention skills and a reduction in suicidal behaviors among at-risk individuals.

## Supporting information

S1 Table Cronbach’s alpha values for the subscales on the included surveys pre – and posttest. (PDF)

S2 Table Individual items with statistically significant improvement on the Willingness to Intervene against Suicide Questionnaire. (PDF)
