# Supplementary material for: The impact of suicide prevention training for nursing assistant students: Knowledge and willingness to intervene
Source: PLoS One. 2025 May 7;20(5):e0323169. doi: 10.1371/journal.pone.0323169 (PMC12057869; doi:10.1371/journal.pone.0323169)
Supplement: S1 Table — (PDF) [file pone.0323169.s001.pdf]

S1 Table 1. Cronbach's alpha values for the subscales on the included surveys pre – and posttest.

Scale	Number of items	Cronbach's Alpha Pre	Cronbach's Alpha Post
**Willingness to intervene against suicide questionnaire**			
Attitudes	12	0.77	0.74
Subjective norms	9	0.79	0.85
Perceived behavioral control	17	0.85	0.84
Intention to intervene	20	0.67	0.81
**Revised facts on suicide quiz**			
Facts and myths about suicide	13	0.55	0.46
Rates and gender differences	8	0.46	0.36

Cronbach alfa values: Poor reliability (below 0.7), Moderate reliability (between 0.7 and 0.8), Good reliability (between 0.8 and 0.9), Excellent reliability (above 0.9).
