# Supplementary material for: The impact of suicide prevention training for nursing assistant students: Knowledge and willingness to intervene
Source: PLoS One. 2025 May 7;20(5):e0323169. doi: 10.1371/journal.pone.0323169 (PMC12057869; doi:10.1371/journal.pone.0323169)
Supplement: S2 Table — (PDF) [file pone.0323169.s002.pdf]

S2 Table 2. Individual items with statistically significant improvement on the Willingness to Intervene against Suicide Questionnaire

Item	Pretest (n = 89)		Posttest (n = 68)		df	95 % Confidence Interval		t	*P*-value	Cohen's d*
	Mean	SD	Mean	SD		Lower	Upper			
**(AT[1])** *Intervening when someone is suicidal would be..*										
Worthless/Valuable	4.34	1.02	4.61	0.92	0.26	-0.04	0.57	1.66	0.04	0.26
Difficult/Easy	1.78	0.95	2.07	1.01	0.28	-0.02	0.59	1.81	0.03	0.29
**(SN[2])**										
It is expected of me that I seek help for someone who is suicidal	3.70	0.93	4.00	0.11	0.30	-0.001	0.58	1.96	0.02	0.31
Most students at my school would intervene	3.40	0.91	3.91	0.87	0.51	0.22	0.79	3.50	<.001	0.56
**(PBC[3])**										
I don't think I can prevent someone from suicide	3.31	0.94	3.69	0.86	0.38	0.08	0.66	2.55	0.005	0.41
I do not know what to say to someone who is suicidal	3.10	0.91	3.41	0.85	0.31	0.02	0.59	2.16	0.01	0.34
I have the interpersonal skills necessary to discuss the presence of suicidal thoughts with a suicidal individual	3.17	0.93	3.64	0.84	0.47	0.18	0.75	3.23	<.001	0.54
I am not confident I know how to intervene with someone who is suicidal	2.87	0.93	3.19	0.73	0.32	0.04	0.58	2.27	0.01	0.36
I do not know where to seek help for someone who is suicidal	3.24	1.15	3.85	3.64	0.61	0.27	0.93	3.62	0.002	0.58
I have access to suicide intervention resources	2.75	1.03	3.73	0.89	0.98	0.67	1.29	6.24	<.001	1.00
I have the skills necessary to clearly convey my concern for the suicidal person	3.10	0.97	3.60	0.83	0.50	0.21	0.79	3.39	<.001	0.54
I am confident I could access	3.41	0.95	3.89	0.81	0.48	0.19	0.76	3.34	<.001	0.53

**Item**	**Pretest (n = 89)**		**Posttest (n = 68)**		**df**	**95 % Confidence Interval**		**t**	***P*-value**	**Cohen's d***
suicide prevention resources										
I believe I am quite knowledgeable about suicide prevention	2.71	1.01	3.30	0.83	0.59	0.29	0.88	3.90	<.001	0.62
	Mean	SD	Mean	SD		Lower	Upper			
**INT[4]**										
Ask the individual if he or she is suicidal	3.32	0.93	3.95	0.88	0.63	0.33	0.92	4.26	<.001	0.68
I would intervene	4.15	0.70	4.38	0.69	0.23	0.002	0.44	1.99	0.02	0.32
Avoid the subject of suicide unless the person who is suicidal brings it up first	3.01	1.11	3.61	1.07	0.6	0.25	0.95	3.42	<.001	0.55
Do nothing; it is none of my business	4.14	0.96	4.51	0.74	0.37	0.09	0.64	2.62	0.004	0.42
Seek help for the suicidal person	3.83	0.82	4.19	0.65	0.36	0.11	0.60	2.94	0.002	0.47
Offer hope that things will get better	3.60	0.92	4.02	0.89	0.42	0.13	0.71	2.87	0.002	0.46
Ask the person if they are having suicidal thoughts or feelings	3.40	0.86	4.20	0.65	0.80	0.55	1.04	6.37	<.001	1.02
Encourage the person who is suicidal to seek help from a suicide crisis hotline	3.96	0.81	4.17	0.68	0.21	-0.03	0.45	1.70	0.04	0.27
Do nothing, assume the person is only seeking attention	4.32	0.95	4.70	0.67	0.38	0.11	0.64	2.80	0.002	0.45
Ignore the situation as it is not my place to intervene	4.35	0.91	4.66	0.58	0.31	0.15	0.65	3.17	<.001	0.51
Tell the person who is suicidal to seek help from a suicide prevention webpage	3.06	1.16	3.45	0.93	0.39	0.04	0.72	2.24	0.01	0.36
Encourage the person to seek help from local resources	3.73	0.82	4.02	0.69	0.29	0.05	0.54	2.41	0.008	0.38

SD: Standard deviation

[1]AT = Attitude; [2]SN = Subjective Norms; [3]PBC = Perceived Behavioral Control; [4]INT = Intention

*Effect size: small (d = 0.2), medium (d = 0.5), and large (d ≥ 0.8).
